# Case I—Chronic Army Diarrhœa

**Published:** 1871-03

**Authors:** 


### Clinical Reports.

### CLINICAL CASES IN MEDICAL WARDS OF MERCY HOSPITAL.

Clinic by PROF. DAVIS. From Notes by S.

MERCY HOSPITAL, *February, 1871.*

CASE 1—CHRONIC ARMY DIARRHŒA.—This patient was in the army, in active service, and endured great hardships. In 1863 he contracted a chronic diarrhœa, which has persisted in spite of every method of treatment which has been tried up to the present time. He is now much emaciated; skin dry and harsh; circulation languid and feeble; tongue pale, but clean and moist; breathing slow and regular. On his admission to the Hospital, the passages from the bowels were copious, but thin, white and frothy, like soapsuds, occurring without pain, and containing no mucus. The urine was abundant, clear, and natural.

While he was living upon an ordinary mixed diet, the bowels would move five or six times a-day, at certain intervals, followed by periods of quiet.

The pathology of these cases is involved in some degree of obscurity. The cases, as they have come under my observation, are capable of arrangement into two classes. One in which the discharges are reddish brown, with some mucus intermixed, some tenderness of abdomen, quick pulse, and dryness and redness of tongue, with feverishness, especially in the afternoon. The *post mortem* examination in these generally shows hypertrophy and ulceration of the glandular structures in the small intentines and colon. The second class of cases is fairly represented by the case now before the class.

The only alterations detected on *post mortem* examinations have been attenuation and atrophy of the mucous membrane of the intestines and atrophy of the liver. This condition is probably the result of the morbid nervous sensibility which causes a perversion of the natural function of the mucous membrane of the intestine, so that instead of imbibition there is constant exudation.

The serous fluid which escapes in this way, together with undigested portions of the food, constitute the discharges.

Treatment.—The bowels could be held in check for a certain length of time by astringents, but they would produce so much bloating and discomfort that the patient would be clamorous for physic in less than twenty-four hours, and at length there would be poured out a copious, profuse evacuation, equal in amount to the several discharges which would have occurred had astringents not been given. Our object should be first to restrict the diet, as far as possible, to those articles which can be digested and absorbed by the stomach and duodenum without leaving any residue to pass through the bowels; secondly, by the use of proper remedies, to overcome, if possible, the morbid and perverted sensibility of the intestinal mucous membrane. For nourishment this patient had been furnished with milk-porridge, in moderate quantity, often enough to afford good support to the system, the quantity to be increased as fast as he is able to appropriate it. At present he is taking about three pints in the twenty-four hours.

For medicine, the ordinary turpentine and laudanum emulsion was ordered to be given, once in six hours at first, and afterward increased to once in four hours. The following prescription—

℞ Bromine,............................xv gtts.

Bromide potass.,.....................ʒiii.

Aqua dist.,..............................℥iiii.

was also directed to be given, in teaspoonful doses, four times a-day. The bromine will, usually in the course of three or four days, change the color of the passages to a bright yellow.

It is useless to attempt to stimulate the liver. There is a suspension of secretion from atrophy, and alteratives administered for the purpose of acting upon this organ would only add to the prostration.

